# Foot–Floor Contact Sequences: A Metric for Gait Assessment in Parkinson’s Disease after Deep Brain Stimulation

**DOI:** 10.3390/s24206593

**Published:** 2024-10-13

**Authors:** Marco Ghislieri, Valentina Agostini, Laura Rizzi, Chiara Fronda, Marco Knaflitz, Michele Lanotte

**Affiliations:** 1 BIOLAB, Department of Electronics and Telecommunications of Politecnico di Torino, 10129 Turin, Italy; marco.ghislieri@polito.it (M.G.); marco.knaflitz@polito.it (M.K.); 2 PolitoBIOMed Lab of Politecnico di Torino, 10129 Turin, Italy; 3 Department of Neuroscience “Rita Levi Montalcini”, University of Turin, 10126 Turin, Italy; l.rizzi@unito.it (L.R.); michele.lanotte@unito.it (M.L.); 4 AOU Città della Salute e della Scienza di Torino, 10126 Turin, Italy; chiarafronda@gmail.com

**Keywords:** DBS, foot–floor contact, gait analysis, locomotion, PD, UPDRS

## Abstract

Digital gait monitoring is increasingly used to assess locomotion and fall risk. The aim of this work is to analyze the changes in the foot–floor contact sequences of Parkinson’s Disease (PD) patients in the year following the implantation of Deep Brain Stimulation (DBS). During their best-ON condition, 30 PD patients underwent gait analysis at baseline ($T_{0}$), at 3 months after subthalamic nucleus DBS neurosurgery ($T_{1}$), and at 12 months ($T_{2}$) after subthalamic nucleus DBS neurosurgery. Thirty age-matched controls underwent gait analysis once. Each subject was equipped with bilateral foot-switches and a 5 min walk was recorded, including both straight-line and turnings. The walking speed, turning time, stride time variability, percentage of atypical gait cycles, stance, swing, and double support duration were estimated. Overall, the gait performance of PD patients improved after DBS, as also confirmed by the decrease in their UPDRS-III scores from 19.4 ± 1.8 to 10.2 ± 1.0 ($T_{0}$ vs. $T_{2}$) (*p* < 0.001). In straight-line walking, the percentages of atypical cycles of PD on the more affected side were 11.1 ± 1.5% (at $T_{0}$), 3.1 ± 1.5% (at $T_{1}$), and 5.1 ± 2.4% (at $T_{2}$), while in controls it was 3.1 ± 1.3% (*p* < 0.0005). In turnings, this percentage was 13.7 ± 1.1% (at $T_{0}$), 7.8 ± 1.1% (at $T_{1}$), and 10.9 ± 1.8% (at $T_{2}$), while in controls it was 8.1 ± 1.0% (*p* < 0.001). Therefore, in straight-line walking, the atypical cycles decreased by 72% at $T_{1}$, and by 54% at $T_{2}$ (with respect to baseline), while, in turnings, atypical cycles decreased by 43% at $T_{1}$, and by 20% at $T_{2}$. The percentage of atypical gait cycles proved an informative digital biomarker for quantifying PD gait changes after DBS, both in straight-line paths and turnings.

## 1. Introduction

Gait alterations are frequent and disabling in Parkinson’s Disease (PD) patients, leading to an increased falling risk [1]. High-frequency Deep Brain Stimulation (DBS) of the subthalamic nucleus (STN) is a neurosurgical therapy that has proven successful in alleviating motor symptoms of patients suffering from advanced PD [2,3,4,5,6,7,8]. The efficacy of DBS for improving motor symptoms was clinically assessed through the Unified Parkinson’s Disease Rating III Scale (UPDRS-III) [9].

Gait analysis can be used to objectively quantify abnormalities in locomotion patterns of PD patients [10,11,12] and their modifications after DBS. To monitor gait, previous studies have employed Inertial Measurement Units (IMU) [13,14], walking mats [15], or foot-worn sensors like foot-pressure insoles and foot switches [16]. To classify motor anomalies in PD, the foot movement is very informative compared to the study of other body segments [14], and investigating the foot-floor contact quality during locomotion can provide unique information about fall risk.

A reliable detection of the gait events for timing the gait cycle, and the detailed study of the foot–floor contact sequence of gait phases, can be obtained through a direct measurement system based on foot-switches [16,17,18]. The PD patients showed an increased percentage of gait cycles with an irregular pattern of foot-floor contact with respect to the controls and these “atypical” gait cycles (e.g., forefoot and flatfoot initial-contact gait cycles) were suggested to be tightly related to an increased fall risk [19].

Independent from the technique used to perform gait analysis and the gait parameters considered, the great majority of the studies focus solely on straight-line walking [10,11,15,20,21], neglecting turnings and curved trajectories. This has been carried out because straight-line walking is more repeatable than curved trajectories. Nevertheless, the walking patterns collected during turnings can be altered even in early PD stages [22,23]. Furthermore, curved walking and turnings induce more gait instabilities and variability compared to straight walking [24] and, hence, are more challenging for patients.

This study aims to fill in the gaps in existing research by assessing the gait performance in PD patients before, at 3 months after subthalamic nucleus DBS neurosurgery, and at 12 months after DBS neurosurgery, by monitoring foot-floor contact sequences during a 5 min walk that includes both straight-line paths and turnings. We hypothesized that motor symptom improvements in PD patients could be quantitatively assessed by evaluating the foot-floor contact sequences, specifically through the analysis of the percentage of “atypical” gait cycles. An improvement in PD motor performance is expected to result in a reduction in the percentage of gait cycles characterized by irregular patterns.

## 2. Materials and Methods

### 2.1. Participants

A total of 60 subjects voluntarily participated in this study. Thirty patients suffering from PD were enrolled at the Stereotactic and Functional Neurosurgery Unit of the University of Turin (Turin, Italy) among those patients eligible for high-frequency (130 Hz) bilateral DBS neurosurgery.

Inclusion criteria were: (i) a diagnosis of PD, according to the UK Brain Bank principles; (ii) a good response to levodopa; (iii) medication-resistant motor fluctuation and dyskinesia; (iv) an age at neurosurgery of under 70 years; (v) the absence of freezing of gait and postural instability unresponsive to pharmacological therapy; (vi) the absence of dementia or severe cognitive impairment, psychiatric, or behavioral disturbances as tested through a standardized battery of cognitive tests assessing reasoning, memory, language, and frontal executive functions [19]; (vii) the absence of abnormalities at cerebral MRI or relevant conditions that increase surgical risk; (viii) the ability to walk independently for a few minutes without walking aids or external support during the pharmacological best-ON time window. The only exclusion criterion was the presence of co-morbidities potentially affecting gait performances, such as knee or hip prostheses. Thirty healthy adults were enrolled among the patients’ caregivers (i.e., PD patients’ wives/husbands or partners) as a control group, excluding those reporting neurological or musculoskeletal disorders potentially affecting gait performance.

This study was conducted in accordance with the Declaration of Helsinki and was approved by the Ethics Committee of A.O.U. Città della Salute e della Scienza di Torino—A.O. Ordine Mauriziano—A.S.L. “Città di Torino” (No. 0092029 approved on 11 September 2018). Written informed consent was obtained from all the subjects involved in this study before data acquisition.

### 2.2. Experimental Protocol and Data Acquisitions

**Figure 1:**
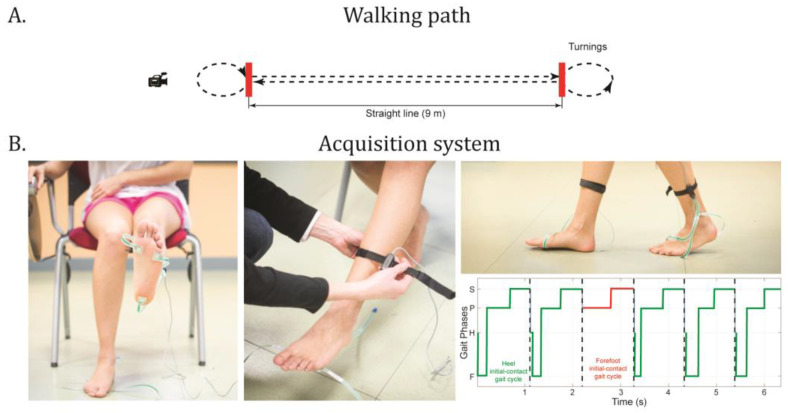
Schematic representation of the walking path (Panel **A**) and acquisition system (Panel **B**). Examples of heel and forefoot initial-contact gait cycles are provided for a representative subject of the sample population with the indication of the four gait phases (H: heel contact, F: flat–foot contact, P: push-off, and S: swing).

The PD patients were always tested during their optimal pharmacological condition ($T_{0}$: medication ON; $T_{1}$: medication ON + DBS ON; $T_{2}$: medication ON + DBS ON). The participants performed a 5 min walk at a self-selected speed, moving back and forth on a 9 m straight-line path. Figure 1A shows a schematic representation of the walking path. The PD patients performed the overground walking task three times: (i) before DBS neurosurgery (baseline, $T_{0}$), (ii) 3 months after DBS neurosurgery ($T_{1}$), and (iii) 12 months after DBS neurosurgery ($T_{2}$), to study both the short- ($T_{1}$) and long-term ($T_{2}$) effects of DBS on walking performance. During the follow-up, the DBS parameters were tailored to the patients’ need to obtain the best possible clinical outcome. In particular, the 3-month and 12-month experimental sessions were conducted during best-ON conditions (i.e., best pharmacological time window and optimal DBS programming settings). The healthy controls performed the walking task only once.

The PD patients were clinically assessed at the Stereotactic Functional Neurosurgery Unit of the University of Turin (Turin, Italy) at baseline ($T_{0}$) and at 12 months after DBS ($T_{2}$). The assessments included the Unified Parkinson’s Disease Rating Scale—Part III (UPDRS-III), the Hoehn and Yahr scale (H&Y), and the Levodopa Equivalent Daily Dose (LEDD).

All the participants were instructed by experimenters to walk along the 9 m straight-line path and then perform a 180-degree turn.

Foot-floor contact sequences were recorded through the STEP32 acquisition system (Medical Technology, Turin, Italy). STEP32 is a multichannel recording system for clinical gait analysis. It can capture up to 16 channels, including foot-switch signals, joint-angle kinematics in the sagittal plane, and electromyographic signals, all synchronized with video recordings. However, in this study, only foot-switch signals were analyzed. Volunteers were equipped, bilaterally, with 3 foot-switches (size: 10 mm × 10 mm × 0.5 mm; activation force: 3 N), fixed beneath the heel, and the first and fifth metatarsal heads of each foot through double-sided adhesive tape. The experimenter determined the optimal foot-switch placement through the palpation of the foot sole and the identification of the main contact points. To ensure the consistency and comparability of the results, the same experimenter performed sensor placement on both the experimental and control populations. The slim design of the foot-switches (0.5 mm thick) ensures that they do not affect gait.

For PD patients, the more affected side was identified based on the side where the disease first manifested, whereas for healthy controls, the dominant side was determined according to the preferred foot to start walking on. Figure 1B shows the placement of the foot-switches and an example of a foot-switch signal acquired during locomotion (sampling rate: 2 kHz) to detect the foot-floor contact sequences. Walking tasks were also simultaneously video-recorded through the built-in camera of the STEP32 system.

All the data acquisitions were performed at the Motion Analysis Laboratory of the Polito^BIO^Med Lab of Politecnico di Torino (Turin, Italy).

### 2.3. Gait Analysis

After the acquisition of the digital signals during walking, the gait performance was quantitatively assessed in terms of: (a) walking speed, (b) turning time, (c) stride time variability, (d) percentage of atypical gait cycles, (e–f) stance and swing phase duration, and (g) double support.

The straight-path time and the turning time were manually estimated by synchronously analyzing gait signals and video recordings and using a stopwatch. The walking speed (ν) was defined as the total distance walked along the straight path (i.e., 9 m) divided by the total time required to go through it. The turning time ($T_{turn}$) was defined as the overall time required by the subject to perform the turnings.

Gait cycles were automatically segmented and classified into typical gait cycles (i.e., with heel initial contact) and atypical gait cycles (i.e., with forefoot and flatfoot initial contact), based on the foot-floor contact sequences detected from the foot-switch signal [16]. Briefly, gait cycles showing the physiological sequence of phases (i.e., Heel contact, Flat foot contact, Push-off, and Swing (“HFPS”)) were classified as “typical” gait cycles. By contrast, gait cycles characterized by the foot-floor sequence “PFPS” (i.e., push off, flat foot contact, push off, and swing), “PS” (i.e., push off and swing), and “FPS” (i.e., flat foot contact, push off, and swing) were classified as “atypical” gait cycles [16]. The percentage of atypical gait cycles was defined as the percentage of gait cycles showing “PFPS”, “PS”, and “FPS” sequences compared to the total number of gait cycles segmented. To assess gait performance improvement, the percentage change in atypical gait cycles with respect to the baseline was computed as ((Follow up time point value−Baseline value)/Baseline value×100).

Stride time variability (${CoV}_{Stride}$) was defined as the coefficient of variation (CoV=standard deviation/mean×100) of the stride durations. From the foot-switch signal, the stance phase duration, the swing phase duration, and the double-support duration were also computed, expressed as a percentage of the Gait Cycle (GC).

The stride time variability (${CoV}_{Stride}$), percentage of atypical gait cycles, stance, swing, and double support were computed for each side (i.e., more-/less-affected side for PD patients and dominant/non-dominant side for healthy controls), separately for straight-line and curvilinear walking.

### 2.4. Statistical Analysis

The differences in anthropometric characteristics between groups (the PD patients—at $T_{0}$ and $T_{2}$—and the healthy controls) were assessed through a two-tailed Student’s *t*-test. A one-way multivariate analysis of variance (*1-way* MANOVA) for repeated measures followed by post-hoc analysis with a Bonferroni adjustment for multiple comparisons was conducted to determine whether there are differences in gait data between groups. The *1-way* MANOVA was conducted considering Group (PD patients and controls) as the between-subjects factor, Body Mass Index (BMI) as the covariate, and all the computed gait parameters (i.e., ν, $T_{turn}$, ${CoV}_{Stride}$, percentage of atypical gait cycles, stance, swing, and double support) as the within-subjects variables. post-hoc analyses with Bonferroni corrections were performed to test differences among groups (PD patients—at $T_{0}$, $T_{1}$, and $T_{2}$—and controls) in all the *1-way* MANOVA within-variables. In all the analyses, the significance level (α) was set as equal to 0.05.

To further evaluate any side-based difference in gait performance, the *1-way* repeated measures MANOVA was performed twice. The first time, only the gait parameters that were extracted from the more affected side of PD patients (dominant side for controls) were considered. The second time, only the gait parameters extracted from the less affected side of PD patients (non-dominant side for controls) were considered. For each population, all the estimated parameters were expressed as mean values and standard errors across the population.

The statistical analysis was carried out using SPSS Statistical Software, version 27.0 (SPSS Inc., Chicago, IL, USA).

## 3. Results

Three out of the thirty PD patients who underwent the gait examinations were then excluded from the final data analysis since they had orthopedic surgery during the follow-up (between $T_{1}$ and $T_{2}$). These patients were excluded from the following analyses to ensure that only PD-related motor deficits were compared, thereby avoiding potential confounding factors from other comorbidities (e.g., hip or knee arthroplasty), which could significantly reduce the comparability of the results. Therefore, 27 PD patients (at three time points) and 30 controls were further analyzed. The anthropometric characteristics of the PD patients (before DBS, and at 12 months after DBS) and the healthy controls enrolled in the study are detailed in Table 1.

No statistically significant differences were detected between the PD patients (before DBS) and the healthy controls for age, weight, and height. A statistically significant reduction in the UPDRS-III motor scale at 12 months after DBS ($T_{0}$: 19.4 ± 1.8; $T_{2}$: 10.2 ± 1.0; *p* < 0.001) was observed, revealing that PD patients clinically improved their motor performance after DBS neurosurgery. Moreover, a statistically significant decrease in LEDD at 12 months after DBS ($T_{0}$: 1354.5 ± 79.9 mg; $T_{2}$: 669.4 ± 65.0 mg; *p* < 0.0005) was found, showing a reduction in the Levodopa Equivalent Daily Dose after DBS surgery.

To evaluate the gait performance, walking speed, turning time, stride time variability, percentage atypical gait cycles, stance, swing, and double support duration, they were assessed during both straight-line walks and turnings. The average gait performance of the PD patients (at $T_{0}$, $T_{1}$, and $T_{2}$) and the healthy controls are represented in Table 2 with the indication of the statistically significant differences between the groups as assessed through the Bonferroni post hoc analysis (indicated by asterisks, daggers, and double daggers).

After adjusting for BMI, there was a statistically significant difference in the gait performances based on Group, F(66, 203) = 1.63, *p* = 0.007, Wilk’s Λ = 0.28, and partial $\eta^{2}$ = 0.34. More specifically, the groups have a statistically significant effect on the turning time (F(3, 89) = 8.27, *p* < 0.0005, partial $\eta^{2}$ = 0.22) percentage of the atypical gait cycles considering both the more affected side (straight-line walk: F(3, 89) = 6.57, *p* < 0.0005, partial $\eta^{2}$ = 0.18; turnings: F(3, 89) = 6.24, *p* = 0.001, partial $\eta^{2}$ = 0.17) and the less affected side (turnings: F(3, 89) = 3.63, *p* = 0.016, partial $\eta^{2}$ = 0.11), the stance phase duration of the more affected side (straight-line walk: F(3, 89) = 3.25, *p* = 0.026, partial $\eta^{2}$ = 0.10; turnings: F(3, 89) = 4.56, *p* = 0.005, partial $\eta^{2}$ = 0.13), and the swing phase duration of the more affected side (straight-line walk: F(3, 89) = 3.57, *p* = 0.017, partial $\eta^{2}$ = 0.10; turnings: F(3, 89) = 4.62, *p* = 0.005, partial $\eta^{2}$ = 0.14). There was no significant effect on the walking speed, stride time variability, and double support based on Group.

**Figure 2:**
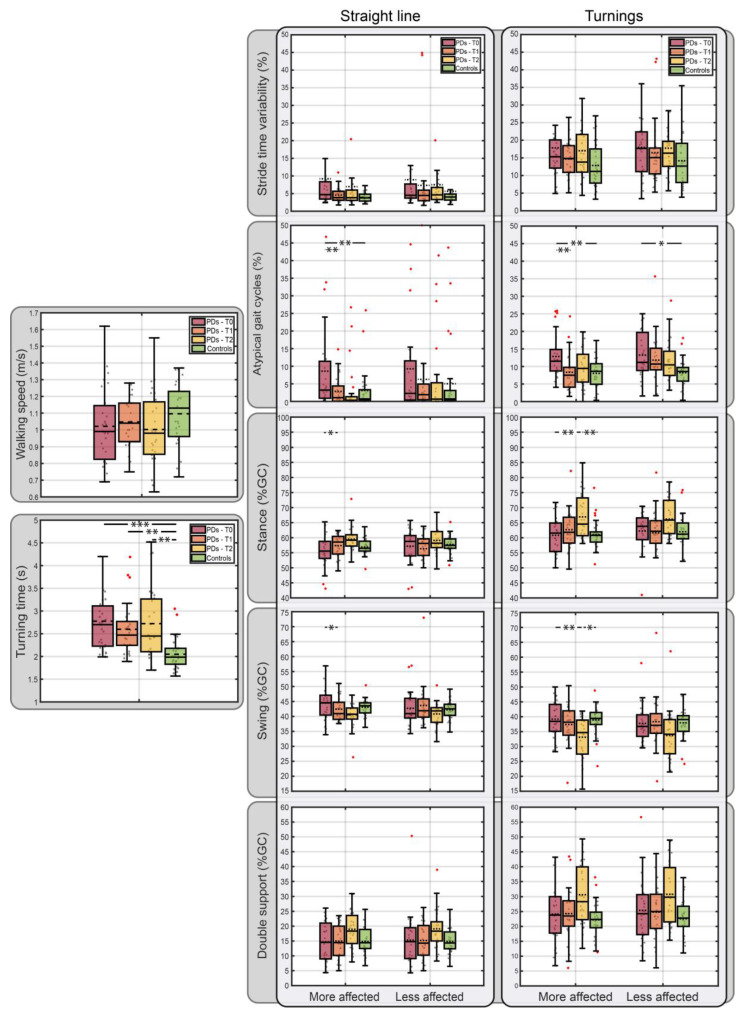
Gait parameters of PD patients (at $T_{0}$, $T_{1}$, and $T_{2}$) and healthy controls. Statistically significant differences (*p* < 0.05) between group mean values are represented by asterisks (* *p* < 0.05, ** *p* < 0.01, *** *p* < 0.001). Data distributions are shown through boxplots representing minimum, 25th percentile, median, mean, 75th percentile, and maximum. Horizontal dotted lines represent mean values.

Figure 2 shows the gait parameters averaged across each sample population with the indication of the statistically significant differences among groups as assessed through the Bonferroni post hoc analysis (indicated by asterisks). For each sample population, Figure 2 shows a standard visualization of central tendency through a boxplot (representing minimum, 25th percentile, median, mean, 75th percentile, and maximum) and the raw jittered data points of each specific individual (scatter plot).

Considering only the more affected (or dominant) side, a statistically significant difference in gait performance (F(36, 243) = 1.99, *p* = 0.001, Wilk’s Λ = 0.47, partial $\eta^{2}$ = 0.23) was detected between the groups. More specifically, the gait performance of the PD patients increased at $T_{1}$ and $T_{2}$, becoming similar to that of healthy controls. No statistically significant group-based differences were detected that considered only the less affected (or non-dominant) side.

## 4. Discussion

This study aimed at assessing gait performance in PD patients before, at 3 months after DBS neurosurgery, and at 12 months after DBS neurosurgery, by monitoring the gaits during a 5 min walk that included both straight-line walking and turnings.

PD gait performances increased at 3 months after subthalamic nucleus DBS neurosurgery ($T_{1}$) and at 12 months ($T_{2}$) after DBS surgery, becoming similar to that of the healthy controls, when considering the tested gait parameters altogether (i.e., velocity, turning duration, stride time variability, percentage of atypical gait cycles, stance/swing duration, and double support) and the walking conditions (i.e., straight-line and curvilinear paths). More specifically, a statistically significant improvement in the PD gait performance after DBS was found when considering the percentage of the atypical gait cycles of the more affected side during both straight-line walks (decreasing from 11.1 ± 1.5% at $T_{0}$ to 3.1 ± 1.5% at $T_{1}$ and 5.1 ± 2.4% at $T_{2}$) and turnings (decreasing from 13.7 ± 1.1% at $T_{0}$ to 7.8 ± 1.1% at $T_{1}$ and 10.9 ± 1.8% at $T_{2}$). In other words, the foot-floor contact sequences of PD at 3 months and at 12 months after DBS became comparable to those of the healthy controls (straight-line: 3.07 ± 1.33%; turnings: 8.05 ± 0.99%), suggesting improvements in motor performance and a potential reduction in fall risk. These improvements align with the overall clinical enhancement observed in PD patients, as suggested by the UPDRS-III scores (decreasing from an average baseline value of 19.4 ± 1.8 points to 10.2 ± 1.0 points at 12 months after DBS).

Considering the percentage change in the atypical gait cycles, an improvement in the PD gait performance is evident. A higher percentage decrease was found at 3 months after DBS with respect to baseline (−72% during straight-line walks and −43% during turnings) than at 12 months after DBS with respect to baseline (−54% during straight-line walks and −20% during turnings). In other words, a higher decrease in the percentage of atypical gait cycles was observed at 3 months after DBS compared to 12 months after DBS. The results demonstrated that PD patients still face difficulties in performing curvilinear trajectories, as suggested by the smaller decrease in the percentage of atypical gait cycles observed during turnings after DBS with respect to baseline.

In a previous study by Ghislieri et al. [19], the percentage of atypical gait cycles was demonstrated to be a valuable biomarker for assessing gait performance in individuals with Parkinson’s Disease. This measure revealed a moderate-to-strong correlation (r = 0.91, *p* = 0.002, 95% CI: [0.59, 0.98]) to the UPDRS-III score, highlighting its potential for providing insights into the severity of motor impairments. The present study further emphasizes the usefulness of this parameter for quantifying gait improvements and evaluates the effectiveness of subthalamic nucleus DBS in advanced PD patients. Notably, the improvement in gait performance was aligned with the clinical enhancement observed following DBS surgery.

The authors focused their attention on two atypical foot-floor contact sequences, the forefoot and flatfoot initial-contact gait cycles, since they hypothesize that the presence of forefoot-strike cycles, and, in particular, “PS” cycles (where the heel never touches the ground during the entire stride), can be related to an increase in fall risk. Depending on the way the turning is approached by the subject (e.g., pivoting on the forefoot or executing a broader curve trajectory), the atypical gait cycles can become more frequent during turnings, even in control subjects. Despite this caveat, the findings of this study emphasize the usefulness of foot-switch recordings, through the estimation of the percentage of atypical gait cycles, to detect changes in PD locomotor control during rectilinear and curvilinear paths, providing valuable insights into the effectiveness of subthalamic nucleus DBS in mitigating motor impairments. In addition, in a previous study, atypical gait cycles were found to be strongly correlated to the Unified Parkinson’s Disease Rating III Scale (UPDRS-III) [9], indicating its potential for the clinical management of PD patients [19]. The possibility of easily collecting foot-switch signals using a portable, lightweight, and low-cost system, after minimal training, highlights the applicability of foot-switch recordings and atypical gait cycle estimation in clinical practice.

Note that this study exclusively considered PD patients who underwent DBS surgery targeting the STN. While this target is widely used, other stimulation sites, such as the Globus Pallidus internus (GPi), are also commonly considered for DBS [25]. Future studies could explore and compare the effects of different DBS targets (STN vs. GPi) on the gait performance in PD patients.

Despite DBS intervention, PD patients revealed higher turning durations ($T_{0}$: 2.8 ± 0.1 s, $T_{1}$: 2.7 ± 0.1 s, $T_{2}$: 2.8 ± 0.2 s) compared to healthy controls (2.1 ± 0.1 s), indicating persistent difficulty in direction changes during walking even after DBS surgery.

In accordance with the previous observation, a longitudinal trend was also observed, in the PD patients’ more affected side, toward an increased stance and decreased swing phase duration, during the turnings. This can be hypothesized to be related to the augmented turning time shown by PD patients, even after DBS.

The previous literature has already established that most PD patients have difficulty with turning, even in the early stages of their disease [26], likely because of the complex interaction of gait with dynamic balance during turning. More specifically, it was reported that turning in PD is characterized by long turning duration (and, consequently, slow speed), a large number of steps [27,28,29], the impaired segmental coordination of rotation (“en-bloc”), a narrow base of support, and decreased postural stability [30,31,32,33,34]. Not surprisingly, PD patients fall five times more than age-matched older adults and they often fall while turning [35].

Based on the results of this study, the assessment of gait performances during turnings, a more task-demanding activity than straight-line walking, proved highly informative given the gait instabilities and alterations induced by curved trajectories in PD patients both before and after DBS surgery. This emphasizes the significance of broadening the scope of experimental protocol designs to encompass curvilinear trajectories, ensuring a more comprehensive and ecological understanding of gait performance.

Some limitations of this study should be acknowledged. The evaluation of gait performance was only conducted during the best-ON pharmacological time window, which may limit the generalizability of the findings to other phases of medication response. Including different pharmacological phases could provide a more comprehensive understanding of the impact of DBS neurosurgery on the gait performance in PD patients. Moreover, while foot-floor contact sequences provide a detailed description of the timing of foot strikes, they do not capture a comprehensive analysis of overall body movements. Future studies could incorporate the assessment of overall body movements, including trunk and upper limb movements, to gain a more comprehensive understanding of how DBS impacts gait performance in Parkinson’s Disease. To better assess the clinical validity of the proposed method, future studies could evaluate the Minimal Clinical Important Difference (MCID) after the careful estimation of the reliability and repeatability of the analyzed gait parameters.

## Figures and Tables

**Table 1:** Anthropometric characteristics of PD patients and healthy controls.

		Sex	Age (Years)	Weight (kg)	Height (m)	UPDRS-III (Best-On Condition)	H&R (Best-On Condition)	Disease Duration (Years)	LEDD (mg)
PD (n= 27)	Before DBS	8 F, 19 M	57.4 ± 1.5	74.4 ± 2.7	1.72 ± 0.02	**19.4 ± 1.8 ^†^**	I–III	11.2 ± 0.6	**1354.5 ± 79.9 ^†^**
	12-mo after DBS		58.6 ± 1.5			**10.2 ± 1.0 ^†^**	I–III	12.3 ± 0.6	**669.4 ± 65.0 ^†^**
Controls (n= 30)		18 F, 12 M	55.0 ± 1.6	74.1 ± 3.4	1.68 ± 0.01	N/A	N/A	N/A	N/A

Parameters’ values are reported as mean ± standard error over sample population. M: males; F: females; UPDRS-III: Unified Parkinson’s Disease Rating Motor Subscale; H&Y: Hoehn and Yahr scale; N/A: not assessed; LEDD: Levodopa Equivalent Daily Dose. Statistically significant differences are represented in bold and through daggers (^†^ *p* < 0.001).

**Table 2:** Gait performance o PD patients (at $T_{0}$, $T_{1}$, and $T_{2}$) and healthy controls.

		PD Patients			Controls	*1-Way* MANOVA
		Before DBS	3 Months after DBS	12 Months after DBS		Group (*p*-Value)
Walking Speed (ms)		1.05 ± 0.04	1.03 ± 0.04	0.99 ± 0.06	1.11 ± 0.03	0.25
Turning Time (s)		2.77 ± 0.13 *	2.68 ± 0.13 ^†^	2.81 ± 0.20 ^‡^	2.05 ± 0.11 *^,†,‡^	**<0.0005**
Stride Time Variability (%)						
Straight-line	More affected	8.48 ± 1.53	4.95 ± 1.53	5.88 ± 2.39	3.89 ± 1.35	0.16
	Less affected	9.92 ± 1.97	9.50 ± 1.97	5.99 ± 3.08	5.62 ± 1.74	0.30
Turnings	More affected	16.74 ± 1.73	14.73 ± 1.73	16.79 ± 2.70	12.65 ± 1.53	0.29
	Less affected	18.75 ± 1.82	17.63 ± 1.82	16.19 ± 2.85	14.05 ± 1.61	0.25
Atypical Gait Cycles (Forefoot and Flatfoot IC) (%)						
Straight-line	More affected	11.07 ± 1.51 *^,†^	3.06 ± 1.51 *	5.09 ± 2.36	3.07 ± 1.33 ^†^	**<0.0005**
	Less affected	10.53 ± 2.62	8.74 ± 2.62	4.18 ± 4.11	5.42 ± 2.32	0.40
Turnings	More affected	13.69 ± 1.12 *^,†^	7.80 ± 1.12 *	10.91 ± 1.76	8.05 ± 0.99 ^†^	**0.001**
	Less affected	13.25 ± 1.22 *	12.28 ± 1.22	9.59 ± 1.90	8.36 ± 1.08 *	**0.016**
Stance (%GC)						
Straight-line	More affected	54.41 ± 0.84 *	57.35 ± 0.84 *	58.52 ± 1.31	56.76 ± 0.74	**0.026**
	Less affected	57.36 ± 1.06	55.62 ± 1.06	58.65 ± 1.66	57.61 ± 0.94	0.38
Turnings	More affected	59.37 ± 1.21 *	62.93 ± 1.21	67.03 ± 1.90 *^,†^	60.56 ± 1.08 ^†^	**0.005**
	Less affected	61.89 ± 1.55	61.12 ± 1.56	66.56 ± 2.42	61.67 ± 1.37	0.28
Swing (%GC)						
Straight-line	More affected	45.70 ± 0.85 *	42.44 ± 0.85 *	41.47 ± 1.33	43.26 ± 0.75	**0.017**
	Less affected	42.51 ± 1.07	44.41 ± 1.07	41.14 ± 1.68	42.34 ± 0.95	0.32
Turnings	More affected	40.62 ± 1.21 *	37.12 ± 1.21	32.91 ± 1.89 *^,†^	39.42 ± 1.07 ^†^	**0.005**
	Less affected	38.09 ± 1.53	38.85 ± 1.53	33.27 ± 2.40	38.28 ± 1.36	0.25
Double Support (%GC)						
Straight-line	More affected	13.98 ± 1.22	14.91 ± 1.22	17.77 ± 1.92	14.62 ± 1.08	0.42
	Less affected	14.28 ± 1.42	15.34 ± 1.42	17.83 ± 2.22	14.63 ± 1.26	0.57
Turnings	More affected	22.75 ± 1.77	23.44 ± 1.77	29.19 ± 2.78	21.62 ± 1.57	0.13
	Less affected	23.86 ± 1.92	24.14 ± 1.92	29.17 ± 3.00	21.98 ± 1.70	0.23

Parameters’ values are reported as mean ± standard error over the sample population (after adjusting for BMI). In PD patients, the more and less affected sides are considered as indicated (in controls, the dominant and non-dominant sides are considered in the correspondent rows). DBS = Deep Brain Stimulation; GC = Gait Cycle. Asterisks; IC = Initial Contact. (*), daggers (†), and double daggers (‡) represent statistically significant differences between groups. *p*-values that are below the significance level α are represented in bold.

## Data Availability

The data and algorithms presented in this study are available from the corresponding author upon reasonable request.
